# I Never Thought I Had “Moral Courage,” until COVID-19 Happened

**DOI:** 10.4269/ajtmh.20-0599

**Published:** 2020-06-24

**Authors:** Maria Elena Bottazzi

**Affiliations:** Department of Pediatrics and Molecular Virology and Microbiology, National School of Tropical Medicine, Baylor College of Medicine, Houston Texas; Texas Children’s Hospital Center for Vaccine Development, Houston Texas; Department of Biology, Baylor University, Waco, Texas

I was in Honduras for the 2019 Christmas holidays when I heard about a pneumonia outbreak in China that potentially resembled SARS-CoV, the virus that caused the 2003 outbreak. Among our team of scientists, we began to wonder if we would be called on to help.

We had begun development of a SARS-CoV vaccine in 2011—one we had worked very hard on, were convinced was promising, and that had even been successfully “manufactured” for human use—but it had been sitting in a regulated freezer in Houston, Texas, since 2016. At that point, my collaborators and I learned that SARS-CoV was no longer a research priority, funding opportunities declined, and the vaccine candidate was unable to progress into the clinical testing phase. This opened my eyes to the realization that without an active crisis, there was no road map to advance vaccines for emergency preparedness.

I always knew we could have another coronavirus outbreak and a vaccine would be needed, although I never imagined such an outbreak could be this serious. And in hindsight, that is what helped affirm the decision that we would not stop testing the stability of our SARS-CoV valuable clinical-grade vaccine candidate.

Four years later, in January 2020, as more news came out of China, I recognized the need to act. “Let’s be proactive in case we need to deploy our vaccine against this mystery virus,” I told our team.

The importance of our vaccine candidate was affirmed after publication of the genetic sequence of SARS-CoV-2 on January 11; it was 80% similar to SARS-CoV. “Advancing our vaccine and securing funding is going to be a no-brainer,” I thought. We only needed a modest investment, around US$3–4 million to bring our vaccine into the clinic.

We started reaching out to partners, peers and others, funding agencies, and media outlets, alerting them that we had a vaccine that could be an important, readily available solution against this new coronavirus.

However, I was wrong.

Responses to our call to action were mixed at best. It did not matter what we said or how much data we or others had on our proven SARS-CoV vaccine approach—one focused on a recombinant protein-based platform widely used for other licensed vaccines and targeting the receptor-binding domain of the viral spike protein. There seemed to be no interest. Our vaccine was not created for the specific virus causing COVID-19, and, thus, traditional funding agencies were not interested. Meanwhile, riskier vaccine technologies with no prior track record as licensed vaccines were being prioritized, with no discussion of how resultant vaccines could be safe and effective while produced at scale, a major focus of our team, or of how they could be produced affordably.

At this point, frustration really started to creep up. Enthusiasm among our scientists started to erode.

Fortunately for me, as an American Association for the Advancement of Science Leshner Fellow in infectious diseases, I had received intensive public engagement and science communication training, and knew how to prepare a plan to communicate the value of our vaccine candidate to myriad audiences. In addition, I work alongside Dr. Peter Hotez, my science partner for the last 20 years. He reminded me about Aristotle’s definition of bravery, and of how we should stand up for what is right, even if we have to do so alone.

This is when I realized I had moral courage and was ready—and equipped—to act and engage. Our engagement took a different dimension, less reactive, more proactive, and organized, which required us to adjust our messaging depending on the audience. For example, my team and I increased our reach not only locally but also internationally. We called upon like-minded global nonprofit organizations such as PATH, a global nonprofit organization dedicated to improving public health that knows what it takes to accelerate safe and affordable vaccines for global use. We called upon those that desire to promote the welfare of others, like Love, Tito’s, the philanthropic arm of the Texas-based Tito’s Handmade Vodka, which was interested in supporting research initiatives in Texas that could have an impact locally and globally during this pandemic.

Today, our effective public engagement and science communication strategy, emphasizing evidence-based science to audiences, is starting to pay off. We continue to forge key strategic alliances, which is validating the scientific integrity of our coronavirus vaccine approach. Our SARS-CoV vaccine as well as a second COVID-19-specific vaccine are finally on the path toward the clinic.

Although the road for these vaccines to be deployed in the community still includes traversing complex clinical development hurdles, including demonstrating that it is safe and effective in humans, this experience has taught me some valuable lessons.

I should always be prepared for stumbling blocks in my career, especially when the evidence-based science I firmly believe in is temporarily shelved. I should never forget to effectively communicate to different audiences. I should never give up when the times get tough.

This will ensure that science is always given another chance—in a way that can not only advance and change my own life but, more importantly, may also save the life of others.

